# Using arm span to derive height: Impact of three estimates of height on interpretation of spirometry

**DOI:** 10.4103/1817-1737.39574

**Published:** 2008

**Authors:** S. K. Chhabra

**Affiliations:** *Cardiorespiratory Physiology, Clinical Research Centre, Vallabhbhai Patel Chest Institute, University of Delhi, Delhi, India*

**Keywords:** Arm span, interpretation, spirometry

## Abstract

**BACKGROUND:**

When standing height required to calculate forced vital capacity (FVC) cannot be measured, it can be derived from arm span using different methods.

**OBJECTIVES:**

To compare three different estimates of height derived from arm span and investigate their impact on interpretation of spirometric data.

**METHODS:**

In a cross-sectional study, 517 subjects aged 7 to 76 years, with various respiratory diseases underwent spirometry. Three estimates of height were obtained from arm span: (a) by direct substitution (Ht_AS_); (b) estimated height (Ht_est_), obtained from the mean arm span:standing height ratio; and (c) predicted height (Ht_pred_), obtained from arm span by linear regression analysis. Predicted values of forced vital capacity (FVC) obtained from these estimates were compared with those obtained from actual standing height (Ht_act_), followed by Bland Altman analysis of agreement in the patterns of ventilatory impairment.

**RESULTS:**

The arm span was 5%-6% greater than the height. The difference increased with increasing height. Ht_AS_ and the FVC predicted from it were significantly greater than the other measures of height and the related predicted FVCs respectively. Compared to Ht_act_, Ht_AS_ gave a misclassification rate of 23.7% in taller subjects (Ht_act_ > 150 cm) and 14.2% in shorter subjects in the patterns of ventilatory impairment. Misclassification rates were 6%-8% with Ht_est_ and Ht_pred_. Agreement analysis showed that FVCs predicted from Ht_pred_ had the best agreement with the FVC predicted from Ht_act_.

**CONCLUSIONS:**

Among several methods of estimating height from the arm span, prediction by regression is most appropriate as it gives least errors in interpretation of spirometric data

Assessment of lung function by spirometry constitutes an important part of diagnostic work-up of a patient with respiratory disease.[1,2] Interpretation requires comparison of observed with normal values that are obtained from prediction equations. Standing height and age are the major determinant variables of forced vital capacity (FVC) and explain most of its variance.[3]

In patients unable to stand straight due to physical disability, structural defects such as kyphoscoliosis or neuromuscular weakness or leg amputation, standing height can be estimated from arm span measurements.[4–10] This may be done by direct substitution by the latter[4–6,10] or by application of a fixed correction factor based on arm span:height ratio[11] or by estimating height from arm span using regression equations.[7–9] For reasons of simplicity, direct substitution by arm span has been favored, both in children[4] and adults.[5,10] The errors introduced by such substitution in interpretation of spirometric data have been reported to be small.[4,6] The recent joint statement of the American Thoracic Society (ATS) and the European Respiratory Society (ERS) has also recommended such substitution.[3] However, the agreement between arm span and standing height has been found to be poor.[8,12] Direct substitution of arm span for height was also questioned recently by Golshan *et al.,*[9] who found estimation from regression models to be superior in healthy subjects. As these three methods may not provide similar estimates of standing height, the predicted values will differ and could have an impact on interpretation. An agreement analysis of predicted vital capacity values obtained from height estimated by different methods from the arm span, as well as an evaluation of the impact of predicted vital capacity values on interpretation of spirometric data in patients suffering from respiratory diseases, has not been done. Therefore, the present study was carried out.

## Material and Methods

The study protocol was approved by the institutional ethics committee. Patients, both children and adults, suffering from different respiratory diseases and referred to the Pulmonary Function Laboratory of our institute were considered for inclusion in the study. The inclusion criteria were an ability to stand erect for measurement of standing height, absence of any spinal and other skeletal abnormality of the limbs, and performance of acceptable spirometry maneuvers as per the ATS recommendations.[1] It was a cross-sectional study. A total of 550 subjects were screened and 517 were found eligible. There were 287 male and 230 female subjects. The age ranged from 7 to 76 years. The purpose of the study was explained and consent taken.

Standing height (actual height, Ht_act_) was measured with a stadiometer in barefoot condition with head in the Frankfurt plane. Arm span (AS) was measured while the patients were asked to stand with their backs against the wall and arms spread in a straight line parallel to the floor. It was measured from the tip of the middle finger of the right hand to the tip of the middle finger of the left hand across the chest at the clavicles. All readings were taken to the nearest 0.5 cm. All the measurements were performed thrice, and the mean was taken and rounded off to the nearest centimeter. Arm span used as a surrogate for height was labeled as Ht_AS_.

Besides Ht_act_ and Ht_AS_, two additional measures of stature were derived. Estimated height (Ht_est_) was obtained by dividing the AS with the mean AS:Ht_act_ ratio. As the AS:Ht_act_ ratio was not different in males and females, the mean of the whole sample was used. Predicted height (Ht_pred_) was obtained by developing a linear regression equation with arm span as the independent variable.

Spirometry was performed on a dry rolling-seal spirometer of the Benchmark model lung function machine (P. K. Morgan, Kent, UK). Maximal expiratory flow volume curves were obtained as per the ATS 1995 recommendations.[1] Three acceptable and at least two reproducible curves were obtained in each subject. The highest values of FVC and FEV_1_ were selected.

Predicted values of FVC were calculated using regression equations for healthy north Indian adults[13] and children.[14] Forced vital capacity (FVC) obtained from Ht_act_ was taken as the reference and labeled as FVC-Ht_act_. Predicted FVCs were obtained from other three measures of stature and labeled as FVC-Ht_AS_, FVC-Ht_est_, and FVC-Ht_pred_. The lower limits of normal (LLN) for FVC were calculated as the difference between the predicted value and 1.645 times the standard error of estimate (SEE). An FEV_1_/FVC <70% with FVC in the normal range was taken to indicate an ‘obstructive disorder.’ An FVC below LLN with a normal FEV_1_/FVC ratio was labeled as ‘restrictive disorder.’ An FEV_1_/FVC <70% with an FVC below LLN was labeled as a ‘mixed disorder.’ An FEV_1_/FVC ≥70% and FVC above LLN was labeled as ‘normal spirometry.’

Statistical analysis was done using SPSS 11.0 and GraphPad Prism 4.01 for Windows. Data was expressed as mean (sd). Comparisons between two groups were carried out using unpaired paired *t* test. Comparison of multiple groups was done using the analysis of variance followed by Bonferroni's multiple comparison test to identify significantly different groups. Coefficients of correlations in bivariate relationships were obtained using the Pearson's correlation test. Agreement between various sets of measurements was evaluated by calculating the kappa value for nominal variables and by the Bland Altman analysis for continuous variables. Kappa values were interpreted as recommended by Byrt *et al.*[15] Linear regression analysis was used to predict Ht_pred_ from the arm span. Statistical significance of differences was assumed at P < .05.

## Results

The mean (sd) age was 37.4 (17.97) and 34.95 (15.38) years in males and females respectively. The descriptive data of standing height (Ht_act_) and arm span (AS) is shown in Table 1. In an overwhelming majority of the subjects (97.8%), the arm span exceeded height by a mean of 9.66 cm and 8.09 cm in male and female subjects respectively. There was no gender difference (P > .05). The difference was 5.9% in males and 5.4% in females.

**Table 1:** Descriptive data of standing height and arm span

	Males (*n* = 287)	Females (*n* = 230)
Mean (sd)age	37.4 (17.97)	34.95 (15.38)
Mean (sd) height (cm)	162.89 (12.38)	150.85 (8.33)
Height range (cm)	107-184	110-168
Mean (sd)arm span (cm)	172.55 (13.99)	158.94 (10.17)
Arm span range	107-197	111-196
Arm span/height	1.059	1.054
Arm span-height difference (5th-95th percentile) (cm)	2 -16.53	1.18 -15.15
Mean (sd) arm span-height difference (cm)	9.66 (4.21)	8.09 (4.61)
Median arm span-height difference (cm)	9.33	8.00
Patients with arm span > height	284	223
Patients with arm span = height	1	0
Patients with arm span < height	2	7

**Figure 1:**
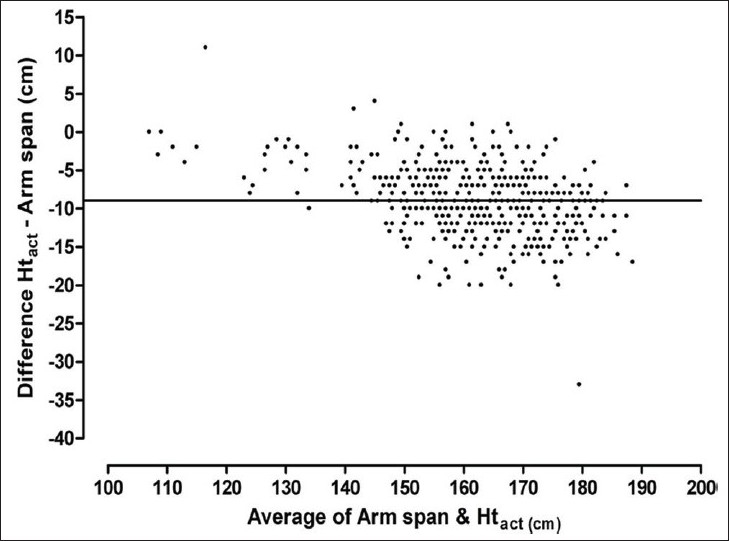
Bland Altman plot of Ht_act_ and arm span. Horizontal line represents the bias

**Figure 2:**
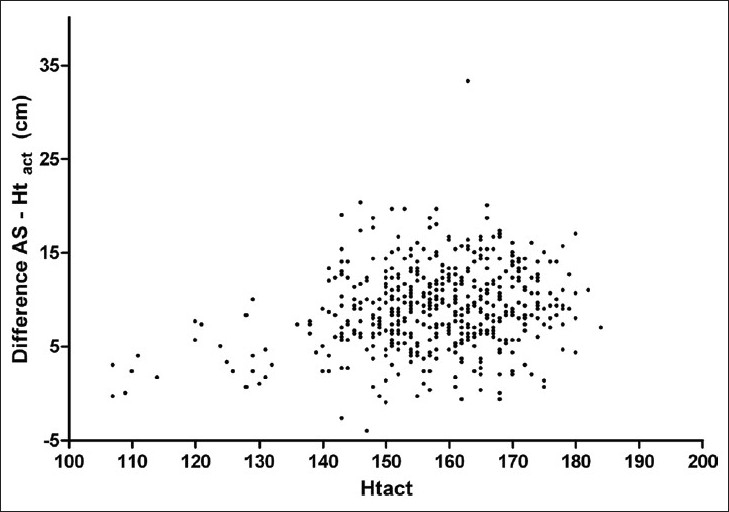
Scatter plot of AS - Ht_act_ against Ht_act_

Ht_act_ and arm span were well correlated (r = 0.95, P < .0001). However, Bland Altman analysis showed a substantial lack of agreement between the two. The mean difference (sd) was –8.93 (4.46) cm with the 95% limits of agreement of –17.68 to –0.19. A definite trend was observed with increasing difference between the two in taller subjects [Figure 1]. A visual inspection of the scatter plot of AS – Ht_act_ against Ht_act_ [Figure 2] revealed that the difference increased with increasing Ht_act_ and reached a plateau at Ht_act_ = 150 cm. The subjects were therefore divided into two groups: group A, Ht_act_ ≥ 151 cm (n = 397); and group B, Ht_act_ ≤ 150 cm (n = 120). With this division, the relationship between AS – Ht_act_ and Ht_act_ no longer remained significant in group A (r = 0.18, P > .05), while it remained significant in group B (r = 0.40, P < .0001). Due to this relationship between AS – Ht_act_ and Ht_act_, subsequent analysis was carried out separately in the two groups.

The linear regression analysis with arm span as the independent variable and Ht_pred_ as dependent variable yielded the following equation in group A: Ht_pred_ = 33.05 + (0.753 × AS) (r^2^ = 0.78, standard expected error, 3.597). In group B, the equation was Ht_pred_ = 30.47 + (0.745 × AS) (r^2^ = 0.89, SEE, 3.51). The four measurements of stature in the two groups are shown in Table 2. ANOVA revealed significant differences (*P* < .0001) among these in both the groups. Between-group comparisons showed that while Ht_AS_ was significantly greater than Ht_act_, Ht_est_, and Ht_pred_ (*P* < .001 for each comparison), the other three were not significantly different on paired comparisons in either group (*P* > .05).

**Table 2:** Comparison of the four measures of stature and predicted FVC obtained from these

Group A		Group B	
	
Measure of stature***	Predicted FVC***	Measure of stature***	Predicted FVC***
Ht_act_ = 162.5 (7.67)	3.56 (0.65)	Ht_act_ = 141.1 (10.33)	2.47 (0.39)
Ht_AS_ = 171.95 (9.00)	3.98 (0.72)	Ht_AS_ = 148.4 (13.03)	2.74 (0.44)
Ht_est_ = 162.5 (8.51)	3.56 (0.66)	Ht_est_ = 141.2 (12.41)	2.47 (0.42)
Ht_pred_ = 162.53 (6.77)	3.56 (0.60)	Ht_pred_ = 141 (9.72)	2.47 (0.37)

Data shown as mean (sd);

*** ANOVA *P* < 0.0001

Predicted FVC (in L) from the four measures of stature revealed significant differences in both the groups (Table 2, ANOVA, *P* < .0001). The FVC predicted from Ht_AS_ was significantly greater than each of the other three FVCs (P < .001), while there were no differences among the latter three estimates on paired comparisons in either group (*P* > .05).

**Figure 3:**
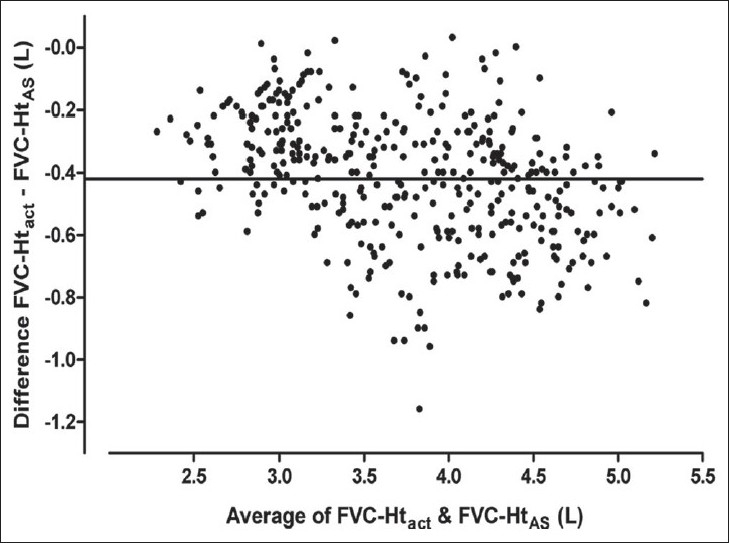
Group A. Bland Altman plot of FVC-Ht_act_ and FVC-Ht_AS_. Horizontal line represents the bias

**Figure 4:**
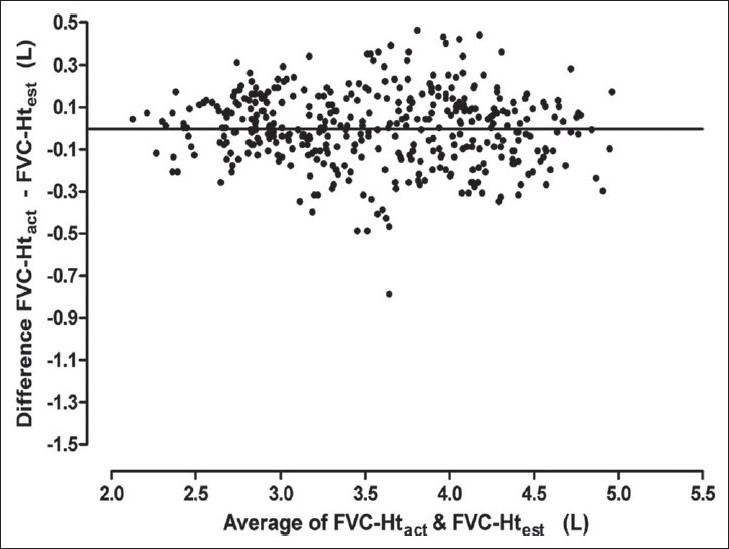
Group A. Bland Altman plot of FVC-Ht_act_ and FVC-Ht_est_. Horizontal line represents the bias

**Figure 5:**
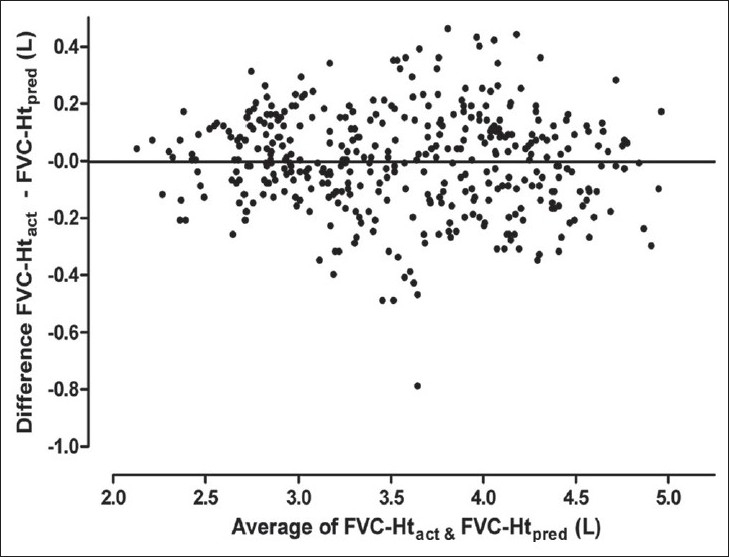
Group A. Bland Altman plot of FVC-Ht_act_ and FVC-Ht_pred_. Horizontal line represents the bias

**Figure 6:**
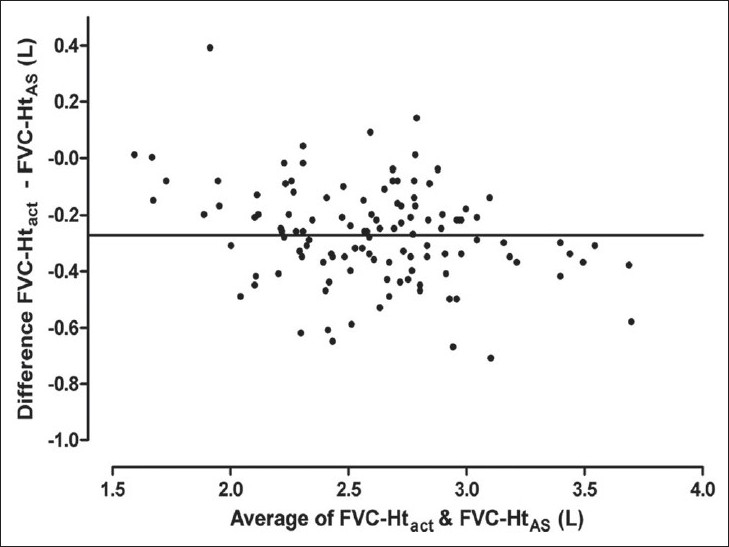
Group B. Bland Altman plot of FVC-Ht_act_ and FVC-Ht_AS_. Horizontal line represents the bias

**Figure 7:**
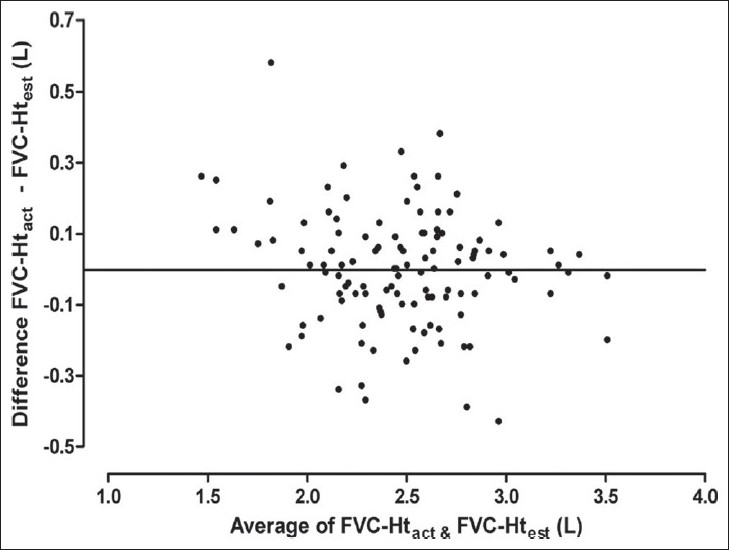
Group B. Bland Altman plot of FVC-Ht_act_ and FVC-Ht_est_. Horizontal line represents the bias

**Figure 8:**
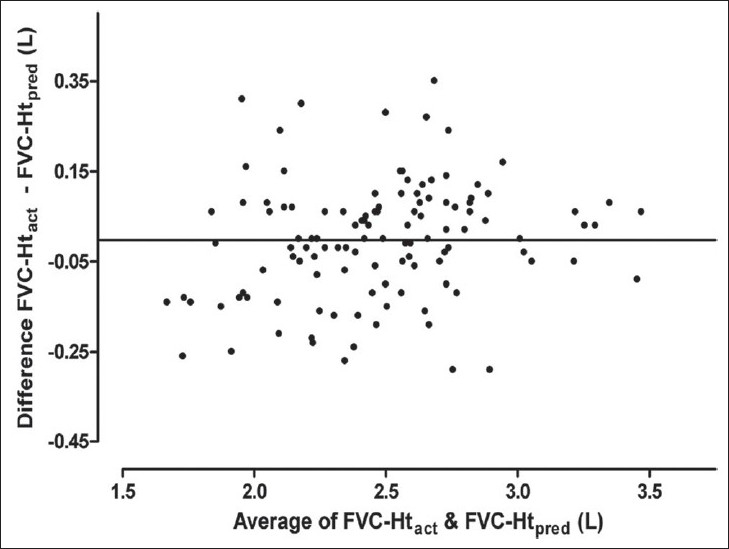
Group B. Bland Altman plot of FVC-Ht_act_ and FVC-Ht_pred_. Horizontal line represents the bias

Results of Bland Altman analysis to examine the agreement between FVC predicted from Ht_act_ and that predicted from the other three estimates of height are shown in Table 3 and Figures 3 to 8. In both the groups, the mean difference between FVC-Ht_act_ and FVC-Ht_AS_ was large with wide limits of agreement. A trend of increasing difference was observed with increasing average of the two [Figures 3 and 6]. On the other hand, the mean difference between FVC-Ht_act_ and FVC-Ht_est_ and between FVC-Ht_act_ and FVC-Ht_pred_ was smaller in both the groups. The scatter around the bias line was uniform [Figures 4 and 5, 7 and 8]. The 95% limits of agreement were narrower and similar between FVC-Ht_act_ and FVC-Ht_est_ and between FVC-Ht_act_ and FVC-Ht_pred_ in group A. In group B, the 95% limits of agreement between FVC-Ht_act_ and FVC-Ht_pred_ were even narrower than those between FVC-Ht_act_ and FVC-Ht_est_.

**Table 3:** Results of Bland Altman analysis of agreement between FVC predicted from Ht_act_ and that predicted from other measures of stature

	Group A		Group B	
		
	Mean difference (bias) and (SD)	95% limits of agreement	Mean difference (bias) and (SD)	95% limits of agreement
FVC-Ht_act_, FVC-Ht_AS_	−0.42 (0.21)	−0.81 to 0.021	−0.27 (0.17)	−0.61 to 0.07
FVC-Ht_act_, FVC-Ht_est_	−0.003 (0.17)	−0.35 to 0.34	−0.001 (0.16)	−0.32 to 0.31
FVC-Ht_act_, FVC-Ht_pred_	−0.003 (0.17)	−0.35 to 0.34	−0.002 (0.13)	−0.26 to 0.25

Unit of FVC: L

The interpretation of spirometry results using the four measures of stature to calculate predicted FVC is shown in Table 4. In group A, out of 397 subjects, 303 were interpreted similarly by Ht_AS_ and Ht_act_, giving a misclassification rate of 23.7% (kappa value for agreement, 0.68, indicating a good agreement). With Ht_est_, 367 subjects were categorized similarly as done with Ht_act_, giving a misclassification rate of 8.4% (kappa value for agreement, 0.89, indicating a very good agreement). Finally, categorization was similar in 373 subjects by Ht_act_ and Ht_pred_, giving a misclassification rate of 6% (kappa value for agreement, 0.88, indicating a very good agreement). In group B, out of 120 subjects, 103 subjects were interpreted similarly by Ht_AS_ and Ht_act_ (misclassification rate of 14.2%; kappa value for agreement, 0.8, indicating a good agreement). Using Ht_est_, 112 subjects were categorized similarly as done by Ht_act_, giving a misclassification rate of 6.7% (kappa value for agreement, 0.90, indicating a very good agreement). Finally, categorization was similar in 110 subjects by Ht_act_ and Ht_pred_, giving a misclassification rate of 8.3% (kappa value for agreement, 0.88, indicating a very good agreement).

**Table 4:** Interpretation of spirometry results using the four measures of stature

	Ht_act_	Ht_AS_	Ht_est_	Ht_pred_
*Group A*				
Normal	184 (46.3)	127 (32)	191 (48.1)	189 (47.6)
Obstructive	80 (20.2)	43 (10.8)	77 (19.4)	77 (19.4)
Restrictive	58 (14.6)	115 (29)	51 (12.8)	53 (13.4)
Mixed	75 (18.9)	112 (28.2)	78 (19.6)	78 (19.6)
*Group B*				
Normal	53 (44.2)	40 (33.3)	55 (45.8)	49 (40.8)
Obstructive	19 (15.8)	15 (12.5)	21 (17.5)	21 (17.5)
Restrictive	30 (25)	43 (35.8)	28 (23.3)	34 (28.3)
Mixed	18 (15)	22 (18.3)	16 (13.3)	16 (13.3)

Figures in parenthesis are column percentages

## Discussion

The results of the study show that arm span was about 5 to 6% greater than the standing height. The difference increased with increasing height and was similar in males and females. Height estimated from a fixed correction factor derived from the arm span:height ratio and/or predicted from arm span using a linear regression equation yielded a value closer to actual standing height than using the arm span as a substitute. Therefore, predicted FVC calculated by using arm span instead of actual height was substantially higher than that obtained using the other two estimates of height. This resulted in large errors in interpretation of spirometric data. Substitution of arm span for actual height resulted in erroneous interpretation in almost one quarter of the subjects taller than 150 cm and in about 15% of the shorter subjects. The errors in interpretation with the other two estimates of height were smaller (6% to 8%) and similar.

The correlation between standing height and arm span is usually excellent.[4,8] Therefore, when actual standing height cannot be measured or is affected by disease as in thoracic vertebral compression due to osteoporosis, and deformity in kyphoscoliosis, arm span has been recommended as a substitute for calculation of the predicted FVC.[2,4,5,10] Parker *et al.*[11] observed that fixed arm span-to-height ratios may also be used to estimate height with reasonable accuracy, but there were errors at extremes of stature. Aggarwal *et al.*[6] observed that height obtained by substitution of arm span or estimation by ratio method resulted in similar errors in interpretation of spirometric data. The errors were however substantial, 16.22% and 14.04% subjects being wrongly classified respectively. They did not evaluate the utility of predicting height from arm span by regression analysis. In a recent study, Golshan *et al.*[9] found that arm span substitution for height over-predicted FVC in healthy subjects compared to FVC calculated from height obtained by regression equations. They did not evaluate the impact of different methods to obtain height on interpretation of spirometry data in patients.

A good correlation does not necessarily mean a good agreement. Torres *et al.*[8] and Hickson and Frost,[12] applying the Bland Altman analysis, observed a poor agreement between the arm span and height even though these correlate well. Hence substitution is not justified. Our observations corroborate these. In addition, we found that height estimated from arm span using a fixed ratio or regression gave similar FVC. This is the first study to report on the impact of these three different estimates of height from arm span on spirometric interpretation in patients with respiratory diseases.

The greater misclassification with direct substitution of arm span for actual height resulted from shifts from normal and obstructive categories to restrictive and mixed categories. This can be explained as arm span yields higher predicted FVC and observed values in patients are more likely to fall below the lower limits of normal and thus get classified as ‘restrictive.’ The misclassification was also greater in taller subjects. Yun *et al.*[16] have reported earlier that growth rates of arm span were greater than those of height in general. In agreement with this, we also observed the difference (arm span – actual height) to increase with height and peaking when the latter reached 150 cm. Thus the over-prediction of FVC on using arm span would be greater in taller subjects. This aspect of the relationship between arm span and standing height was not examined in earlier studies.[6,9] The misclassification has significant clinical implications. There is a risk of wrongly labeling a normal subject as abnormal, as well as overestimating the degree of impairment in those with a true restrictive pathology. Therefore, the practice of substituting arm span for height cannot be recommended.

The FVC predicted by the other two estimates of height was closer to that predicted from actual height, and thus overall agreement in interpretation of spirometry was also very good. Yet the limits of agreement between FVC obtained from these estimates and that obtained from actual height were fairly wide on Bland Altman analysis and therefore, the clinician needs to be careful in interpretation of spirometric data using estimated heights, especially when the values are closer to the lower limits of normal. From the results of this study, it appears that when actual height cannot be obtained, either of the two measures may be used in taller subjects. In shorter subjects, however, height predicted from arm span using regression agrees more closely with actual height than does that estimated using a fixed ratio and is therefore recommended.

The study has limitations. It was not blinded. The population of the study was divided into two groups after the study was completed. This, however, was not possible before the study as the relationship between (arm span – height) and height was not known. Further, generalization to other ethnic backgrounds may not be done as arm span and height correlations have been shown to be different among different ethnic groups. Similar studies therefore need to be carried out in other populations.

In conclusion, when actual height cannot be measured, prediction from arm span using regression analysis appears to be the most appropriate method as it predicts vital capacity closer to the actual and gives least errors in interpretation of spirometric data in patients with respiratory diseases. Direct substitution of arm span for height is not recommended as it yields greater than true predicted vital capacity, resulting in unacceptably high rates of misclassification of spirometric data.
